# Patterns of Cerebrovascular Accidents in Antiphospholipid Syndrome

**DOI:** 10.7759/cureus.65243

**Published:** 2024-07-24

**Authors:** Uthayanila Pandian, Arun K, Raji Rajesh Lenin, Ajay Dev, JS Kumar

**Affiliations:** 1 General Medicine, SRM Medical College Hospital and Research Centre, SRM Institute of Science and Technology, Kattankulathur, Chengalpattu, IND; 2 General Medicine, Karpaga Vinayaga Institute of Medical Sciences, Chennai, IND; 3 Division of Medical Research, Faculty of Medicine and Health Sciences, SRM Medical College Hospital and Research Centre, SRM Institute of Science and Technology, Kattankulathur, Chengalpattu, IND

**Keywords:** transesophageal echo, facial palsy, young stroke, cerebro vascular accidents, antiphospholipid syndrome

## Abstract

Antiphospholipid syndrome (APS) is an autoimmune disease that primarily affects young adults. It is characterized by the development of antiphospholipid antibodies (APL) and a wide range of macro- and microvascular symptoms. The primary causes of morbidity and mortality in APS are cardiovascular events. Subclinical atherosclerosis and cardiovascular events are associated with high-risk APL profiles, particularly with the presence of lupus anticoagulant and triple APL positivity (all three APL subtypes), co-existence with systemic lupus erythematosus (SLE), and traditional risk factors like smoking, hypertension, obesity, and hyperlipemia. We present a case series involving three female stroke patients with APS. This series highlights the importance of immunological profiles in all stroke patients.

## Introduction

The antibody-mediated acquired thrombophilia known as antiphospholipid syndrome (APS) is typified by recurrent venous or arterial thrombosis and/or pregnancy-related morbidity. Catastrophic APS refers to a potentially fatal, quickly progressing thromboembolic illness that affects three or more organs at once [1]. Widespread thrombosis, which can impact the heart and brain among other organ systems, is a hallmark of acute peripheral thrombosis. APS frequently manifests as cerebral artery and venous thromboses, which often result in ischemic and hemorrhagic stroke [2]. APS raises the risk of stroke through a variety of pathways, such as inflammation and hypercoagulability. To achieve optimum results in the short- and long-term, doctors treating such patients need to take into account these mechanisms in addition to others [3]. Timely detection of stroke and its cause is essential to prevent morbidity and death and to start the right treatment as soon as possible [4]. The need for a detailed history, physical examination, comprehensive evaluation, and clinical suspicion in making a diagnosis is emphasized in this case series. It also covers the variations in treatment depending on the kind of cerebrovascular trauma.

## Case presentation

Case report 1

**Figure 1:**
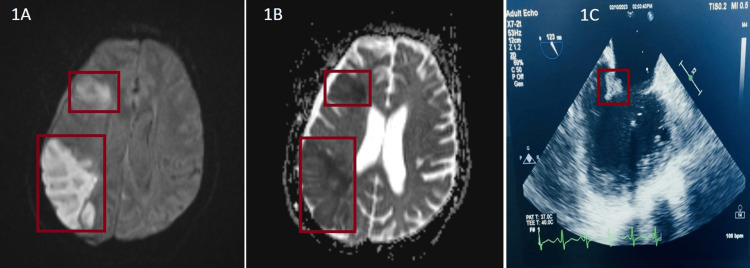
MRI brain and TOE findings 1A: MRI brain showing diffusion restriction in right frontal and parietal region suggestive of acute infarct. 1B: MRI brain showing low ADC suggestive of acute infarct in right frontal and parietal region. 1C: TOE showing verrucous mass under the surface of PML with absent independent motion ADC: apparent diffusion coefficient; MRI: magnetic resonance imaging; PML: posterior mitral leaflet; TOE: transoesophageal echocardiogram

A 37-year-old female patient with no known comorbidities presented with complaints of weakness of the left upper limb and slurring of speech for the past five days. Neurological examination showed reduced motor power in the left upper limb (both proximal and distal muscle) with seventh cranial nerve upper motor neurons (UMN) type palsy on the left side. Other cranial nerves examination and cerebellum and sensory examination were normal. A cardiovascular system examination revealed a pansystolic murmur at the mitral area with radiation to the axilla. CT brain was taken, which showed a hypodense area in the right frontal gyrus and temporoparietal lobe involving the right middle cerebral artery (MCA) territory. MRI brain showed an acute infarct in the right frontal and right parietal regions. The 2D echo (transthoracic) showed thickened anterior and posterior mitral leaflet with the fusion of commissures and mitral valve orifice of 1.8 square cm with fixed posterior leaflet, severe mitral regurgitation, and dilated left atrium (5 cm) with normal left ventricular (LV) systolic function [Ejection fraction (EF): 64%] and mild pulmonary hypertension (Figure 1).

A diagnosis of cardioembolic stroke secondary to rheumatic heart disease (RHD) with mitral stenosis (MS) and regurgitation (MR) was made, and the patient was started on anticoagulation therapy. Since 2D echo showed the presence of RHD with MS and MR, the patient was planned for transoesophageal echo and also worked up for stroke in young patients. Holter monitoring showed no atrial fibrillation. The patient was started on anticoagulation given multiple territory involvement of brain parenchyma, dilated left atrium, and elevated peak A’-wave on the tissue Doppler imaging (PA-TDI). Further evaluation showed antiphospholipid antibody (APLA) triple positivity-b2-glycoprotein (both IgG- and IgM-positive); anticardiolipin (both IgG- and IgM-positive); lupus anticoagulant detected [patient PTT: 52.4, dilute Russell viper venom time (dRVVT)-screening positive]; antinuclear antibody (ANA, strong positive); pattern-granular nucleus; anti-Ro52 strong positive; anti-dsDNA- and anti-SM-negative; C3, C4 normal levels; erythrocyte sedimentation rate (ESR) elevated; and C-reactive protein (CRP) positive.

Based on the value of the low platelet count, thrombocytopenia was observed in this patient. A transesophageal echo showed a verrucous heterogeneous mass under the surface of a posterior mitral leaflet of 1 cm in size, with mobile edges and the absence of independent motion. There was an impression of severe MR with vegetation on the posterior mitral leaflet in the presence of nonbacterial thrombotic endocarditis (NBTE). The patient was further evaluated for infective endocarditis and worked up for other connective tissue disorders. Blood culture sensitivity from three different sites taken six hours apart three times showed no growth of organisms, and the patient had no fever episodes or external markers of infective endocarditis. She was started on low-molecular-weight heparin (Inj. LMWH) for five days bridged with oral vitamin K antagonist on the third day and started on a single antiplatelet tablet of aspirin 75mg and hydroxychloroquine 200mg BD. She was taken up for mitral valve replacement surgery and a biopsy of the excised mitral valve revealed fibrosis, neovascularization, and vegetations with fibrin-platelet thrombi and evident inflammatory cell infiltration suggesting the presence of Libman-Sacks endocarditis in the combined stage of active and healed lesion.

Case report 2

**Figure 2:**
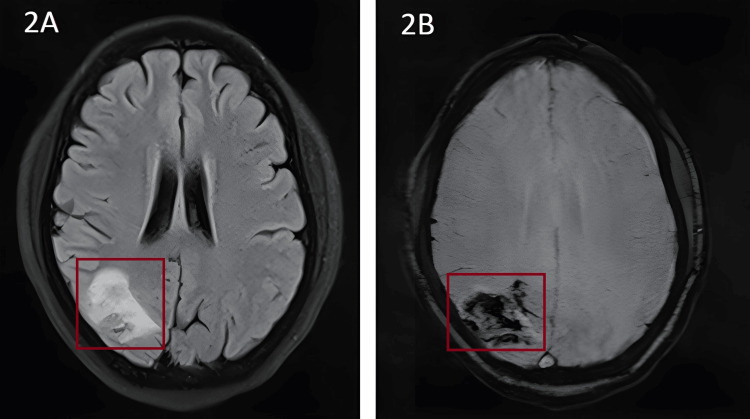
MRI brain findings 2A: MRI brain T2 FLAIR showing heterogeneously hyperintense area involving cortical and subcortical regions of the right parieto-occipital region. 2B: MRI brain SWI showing blooming suggestive of hemorrhagic venous infarct in the same area FLAIR: fluid-attenuated inversion recovery; MRI: magnetic resonance imaging; SWI: susceptibility-weighted imaging

A 25-year-old female with no known comorbidities presented to the casualty with complaints of sudden diminution of vision since morning and c/o headache for seven days. She had no significant past medical history. Her central nervous system examination was completely normal except for visual field acuity of 2/60 with normal color vision in the bilateral eye. An ophthalmological examination was normal in both eyes. CT brain showed acute infarct in the right occipito-parietal region with punctate foci of hemorrhage and thrombosed cortical vein and well-defined hypo-intensity in the right medial temporal lobe (Figure 2).

Routine blood investigations, 2D echo, and chest X-ray were normal. Since the patient was very young, with no known comorbidities, and presenting with CVT, a prothrombotic workup was done, and the APLA profile showed the following findings: antiphospholipid: IgM elevated (17.3); IgG normal, beta 2 glycoprotein - IgM positive (3.14); IgG negative, lupus anticoagulant was not detected; anticardiolipin: IgM negative; IgG negative, ANA profile-negative, anti-thrombin and serum homocysteine normal. A final diagnosis of primary APS presenting as acute CVA with cortical venous thrombosis causing sudden diminution of vision was made and the patient was started on LMWH injection and statins, and bridging was done with acitrom; after three days to maintain target INR at 2.5-3.0. The patient was followed up after one week on anticoagulation and showed visual acuity of 6/9 in bilateral eyes.

Case report 3 

A 24-year-old female patient with no known comorbidities arrived at the emergency room complaining of a deviation of the angle of her mouth to the left side and slurring of speech during the previous 24 hours. There was no documented history of fever, recent infection, skin lesions, or cold exposure. Upon additional questioning, the patient revealed episodes of giddiness, nausea, and vomiting beginning five days before the onset of facial weakness. Of note, there was no reported history of focal limb weakness or sensory disturbances, and the patient denied any head trauma or illicit drug use. The patient had undergone an initial CT scan five days earlier during the onset of giddiness, which returned negative results.

At the presentation, the patient remained conscious and alert, without fever, with a blood pressure of 130/70 mmHg and a heart rate of 78 beats per minute. A neurological examination revealed no objective motor or sensory abnormalities in either the upper or lower limbs but did indicate a positive Babinski sign on the right plantar reflex. Nuchal rigidity was notably absent. Cranial nerve examination demonstrated right facial paralysis of lower motor neuron type, characterized by the loss of forehead crease on the right side, inability to raise the eyebrow on the right side, and deviation of mouth angle to the left side. A positive Bell's phenomenon was observed on the right side. Other cranial nerve examinations yielded normal results. Cerebellum examination revealed cerebellar ataxia and swaying to the right during tandem walking, with no presence of nystagmus or dysdiadochokinesia. The patient had received initial treatment five days earlier, consisting of normal saline, ondansetron, promethazine, and betahistine, which successfully alleviated symptoms of giddiness. Initial investigations were all within normal ranges.

**Figure 3:**
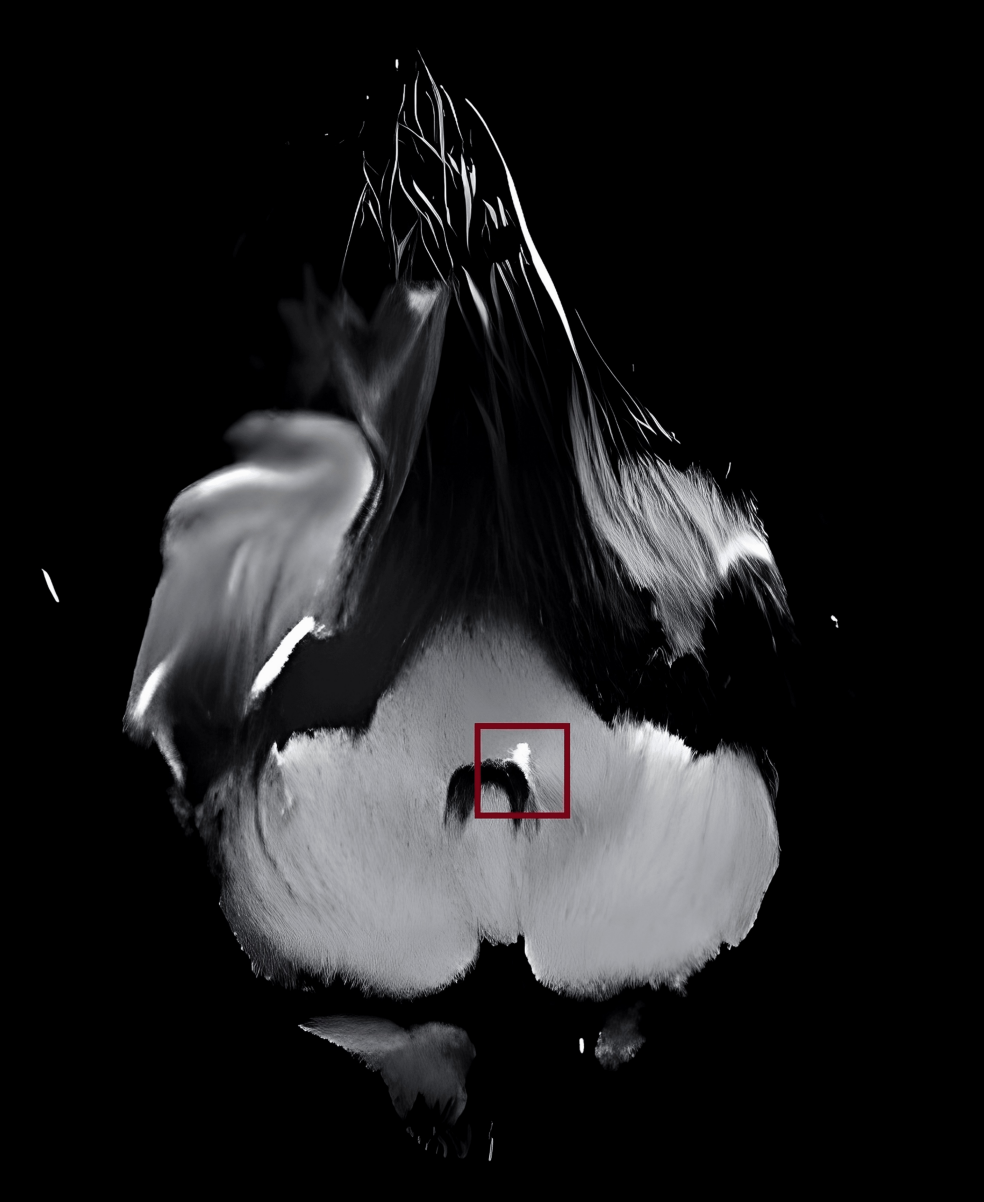
Diffusion-weighted MRI showing hyperintense foci at the junction of left lateral pontine tegmentum and left middle cerebellar peduncle MRI: magnetic resonance imaging

An electrocardiogram yielded normal results. Given the inability to definitively rule out acute ischemic stroke through CT alone, a plain MRI of the brain was performed, which revealed a small non-hemorrhagic lacunar infarct at the junction of the middle cerebellar peduncles (MCP) and lateral pontine tegmentum (Figure 3). The patient was further evaluated for procoagulant states, which showed the following results: APLA: IgM positive; beta 2 glycoprotein - IgM positive; lupus anticoagulant was not detected; and anticardiolipin and ANA were both negative. The patient was started on Inj. LMWH for five days bridged with oral vitamin K antagonist on the third day and was also started on single antiplatelet T. aspirin 75mg and T. hydroxychloroquine 200mg BD.

Table 1 shows the laboratory results and autoantibody profiles of the patients.

**Table 1:** Laboratory results and autoantibody profiles of the patients APLA: antiphospholipid antibody; ANA: antinuclear antibody; CRP: C-reactive protein; ESR: erythrocyte sedimentation rate; WBC: whole blood count

Laboratory findings and autoantibody profiles		
Case 1: Investigation	Result	Reference value
WBC count	3500/mm^3^	4000-110,000/mm^3^
Platelet count	90,000/mm^3^	150,000-400,000/mm^3^
APLA profile		
1. Beta2-glycoprotein	IgG and IgM positive	
2. Anticardiolipin antibody	IgG and IgM positive	
3. Lupus anticoagulant	Detected	
ANA	Positive	
Anti-Ro52	Positive	
C3, C4	Normal	
ESR	Positive	
CRP	Positive	
Case 2: Investigation	Result	Reference value
WBC count	6200/mm^3^	4000-110,000/mm^3^
Platelet count	1,90,000/mm^3^	150,000-400,000/mm^3^
APLA profile		
1. Beta2-glycoprotein	IgM positive, IgG negative	
2. Anticardiolipin antibody	IgM and IgG negative	
3. Lupus anticoagulant	Not detected	
ANA	Negative	
Serum homocysteine	7 micromol/L	5-15 micromol/L
Case 3: Investigation	Result	Reference value
WBC count	5500/mm^3^	4000-110,000/mm^3^
Platelet count	1,50,000/mm^3^	150,000-400,000/mm^3^
APLA profile		
1. Beta2-glycoprotein	IgM positive, IgG negative	
2. Anticardiolipin antibody	IgM and IgG negative	
3. Lupus anticoagulant	Not detected	
ANA	Negative	

## Discussion

It has been established that APS can cause CVA, particularly in people without traditional cardiovascular risk factors [5]. APL antibodies are found in about 14% of stroke patients, according to some recent research, and it has been suggested that one in five strokes in patients under 45 years may be related to APS. Compared to other forms of thromboses, the recurring risk of stroke in individuals with APS has not received much attention; still, a cumulative risk of 14% for brain ischemia after 10 years has been documented [6]. APLA makes its way through the heart's complex network of passageways and into the brain's stronghold. Vitamin K antagonists and antiplatelets (low-dose aspirin) can be used to treat APS-induced cerebral artery thrombosis, which typically affects a single region. The brain is reached through the heart by APS, which causes CVA through a common source of embolism (cardiac valves/proximal aorta) [7]. This can affect both bilateral hemispheres and multiple artery territories. If the involvement of the valve causes insufficiency or embolism, vitamin K antagonist treatment and valve replacement may be considered [8].

APS raises the risk of stroke through several factors, including hypercoagulability, inflammation, accelerated atherosclerosis, and cardiac symptoms. Although the precise underlying pathophysiology of APS is still unknown, it is thought that underlying genetics in the context of a triggering event (such as surgery, trauma, or infection) is crucial to the development of the illness [9]. Even though guidelines for primary and secondary prevention are always evolving, it is best to treat each case separately to attain optimal outcomes [10]. Further randomized controlled trials need to be conducted to draw further conclusions about the constantly evolving consensus criteria. For the time being, the choice of primary and/or secondary preventive medicines, as well as their kind, will remain a patient-centered one that necessitates careful test result interpretations and inputs from multiple specialties (neurology, hematology, and rheumatology) [11].

## Conclusions

Young patients presenting with an idiopathic stroke should be evaluated for underlying immunological or hematological diseases. They should also undergo testing for possible nonbacterial endocarditis with vegetations causing cardioembolic stroke. This case report emphasizes the importance of transthoracic and transesophageal echocardiography in all APS patients as an initial evaluation as well as the need for a thorough central nervous system examination in any patient with facial palsy. Treatment of the underlying condition, surgical removal of vegetation if indicated, starting anticoagulation, and careful follow-up are important for the successful treatment of this condition. Since the prevalence of valve involvement is higher in systemic lupus erythematosus (SLE)-APS than in primary APS, all APS patients should be screened for SLE.
